# Predicting Dose-Range Chemical Toxicity using Novel Hybrid Deep Machine-Learning Method

**DOI:** 10.3390/toxics10110706

**Published:** 2022-11-18

**Authors:** Sarita Limbu, Cyril Zakka, Sivanesan Dakshanamurthy

**Affiliations:** 1 Lombardi Comprehensive Cancer Center, Georgetown University Medical Center, Washington, DC 20057, USA; 2 Faculty of Medicine, American University of Beirut Medical Center, Beirut 1107 2020, Lebanon

**Keywords:** chemical toxicity, machine-learning method, artificial neural network, convolutional neural network, fast-forward neural network, deep learning, binary toxicity, multiclass toxicity, categorical toxicity

## Abstract

Humans are exposed to thousands of chemicals, including environmental chemicals. Unfortunately, little is known about their potential toxicity, as determining the toxicity remains challenging due to the substantial resources required to assess a chemical in vivo. Here, we present a novel hybrid neural network (HNN) deep learning method, called HNN-Tox, to predict chemical toxicity at different doses. To develop a hybrid HNN-Tox method, we combined two neural network frameworks, the Convolutional Neural Network (CNN) and the multilayer perceptron (MLP)-type feed-forward neural network (FFNN). Combining the CNN and FCNN in the field of environmental chemical toxicity prediction is a novel approach. We developed several binary and multiclass classification models to assess dose-range chemical toxicity that is trained based on thousands of chemicals with known toxicity. The performance of the HNN-Tox was compared with other machine-learning methods, including Random Forest (RF), Bootstrap Aggregation (Bagging), and Adaptive Boosting (AdaBoost). We also analyzed the model performance dependency on varying features, descriptors, dataset size, route of exposure, and toxic dose. The HNN-Tox model, trained on 59,373 chemicals annotated with known LD50 and routes of exposure, maintained its predictive ability with an accuracy of 84.9% and 84.1%, even after reducing the descriptor size from 318 to 51, and the area under the ROC curve (AUC) was 0.89 and 0.88, respectively. Further, we validated the HNN-Tox with several external toxic chemical datasets on a large scale. The HNN-Tox performed optimally or better than the other machine-learning methods for diverse chemicals. This study is the first to report a large-scale prediction of dose-range chemical toxicity with varying features. The HNN-Tox has broad applicability in predicting toxicity for diverse chemicals and could serve as an alternative methodology approach to animal-based toxicity assessment.

## 1. Introduction

Humans are exposed to thousands of potentially toxic chemicals, including environmental chemicals, such as industrial chemicals, microplastics, nanoplastics, food products, solvents, air pollutants, fertilizers, pesticides, insecticides, carcinogens, drugs, metals/metalloids, PFOS, burn pits, and other hazardous chemicals. Factors affecting the degree of toxicity are the route of exposure (oral, dermal, inhalation, and injection), duration of exposure, a dose of the chemical, age, gender, and health condition. The conventional in vivo and in vitro tests for finding the toxicity of chemicals are resource intensive. In this context, in silico toxicity prediction [1] is gaining popularity as a quick and inexpensive alternative that eliminates the need for further animal testing, which is controversial due to ethical concerns.

Toxicity predictions could be quantitative, predicting the quantity of chemical required for the adverse outcome or qualitative, predicting binary endpoint (such as whether the chemical is toxic or nontoxic), or ordinary, predicting the categorical endpoint (such as high, moderate, and low). Several computational models are developed using various machine-learning algorithms for various toxicity endpoints [2,3,4]. To predict acute oral toxicity (LD50), regression models are developed with a confined applicability domain to improve prediction accuracy [1,2,3,4]. Chavan et al. used the k-Nearest Neighbor (KNN) method to predict the acute toxicity of chemicals with 79.17% accuracy [5]. Cherkasov et al. predicted the antibacterial activity of chemicals with 93% accuracy using the Artificial Neural Network (ANN) method for the Quantitative Structure–Activity Relationship (QSAR) model [6]. Zhang et al. reported 70% accuracy on binary models for predicting chemical carcinogenicity using an ensemble of Support Vector Machine (SVM), Random Forest (RF), and Extreme Gradient Boosting (XGBoost) machine-learning methods [7]. Tanabe et al. reported an accuracy of 70% using SVM modeling and improved it to 80% by developing models on the chemical subgroups based on their structure [8]. Li et al. developed multiclassification models using SVM, RF, decision tree, k-Nearest Neighbor (kNN), and Naïve Bayes for predicting the categorical toxicity of chemicals, with overall accuracy ranging from 42% to 83% and from 25.1% to 89.9% for external validation set I and II, respectively [9].

Deep neural network (DNN) architecture is an ANN with more than one hidden layer. Convolutional Neural Network (CNN) is a class of deep neural network more suitable for image processing tasks, such as visual data recognition [10,11,12]. The DNN has been successfully applied in many areas, including speech recognition [10] and image recognition [11]. The strength of DNN has been demonstrated in toxicity prediction by Mayr et al. [13] by achieving AUCs between 0.79 and 0.94 for different cell-based assay training datasets. Dahl et al. used the DNN for compound activity prediction [14]. Deep learning has also been used in image-based toxicity prediction [15,16] and drug-induced liver injury prediction [17]. The deep-learning-based multiclass model, developed by Xu et al., predicts 95.5% and 96.3% accuracy on test sets I and II, respectively [2]. Notably, the DNN performs better with larger datasets [18]. The success of deep learning models proves that they have great potential in the field of toxicity prediction.

Taken together, several computational and machine-learning methods are reported in the literature for chemical toxicity prediction [3,4,5,6,7,8,9,10,11,12,13,14,15,16,17,18]. However, to predict toxicity reliably, these machine-learning models require large, diverse chemical datasets to train the model. The more dimensions or diversification in the model desired, the larger the dataset needed for training the model. Otherwise, an overfitting problem exists. Notably, it is imperative to test the predictions with various training dataset sizes, doses, routes of exposure, and descriptors to test the dependency and performance variations in the neural network models. Further, comprehensive dose range multiclassification models and class imbalance among the datasets are important to reliably assess the toxicity of diverse chemicals in a dose-dependent manner. Thus, a highly reliable model is needed to predict the chemical toxicity of diverse chemicals in a dose-dependent manner. To fill this gap, here, we present a novel hybrid neural network (HNN) method called HNN-Tox that predicts chemical toxicity at different dose ranges with varying features. We used 92,322 diverse chemicals with known multispecies, including rat and mouse toxicity data, to develop various classification models. The HNN-Tox presented the best or most optimal predictive performance for most datasets compared to the other machine-learning methods.

## 2. Materials and Methods

### 2.1. Training and Test Dataset and Feature Attributes

We collected thousands of chemicals annotated with experimentally known toxic dose data from different samples, attributes, properties, and sources to build the HNN-Tox and other machine-learning models. We used the following data sources: (i) ChemIDplus, (ii) Toxin and Toxin Target Database (T3DB), (iii) Environmental Protection Agency (EPA), and (iv) Tox21 Challenge. Models were developed for each dataset and the prediction capability was assessed. The T3DB and National Toxicology Program (NTP) datasets were used as an external validation set to test the models’ predictive ability.

#### 2.1.1. Binary Classification Models

Datasets were annotated with toxicity classification at various LD50 cutoffs (250 mg/kg, 500 mg/kg, 750 mg/kg, and 1000 mg/kg) to compare the impact of various dose cutoffs and resulting class imbalance on the predictive toxicity performance of the models.

##### ChemIDplus Dataset

CAS registry numbers (CASRNs) were obtained from the ChemIDplus database at ftp://ftp.nlm.nih.gov/nlmdata/.chemidlease/ accessed on 12 October 2019. These CASRNs were used to retrieve the LD50 data from the ChemIDplus using the open stream() method of URL class in Java’s java.net package. Through this Java package, we retrieved a total of 386,620 chemicals with CASRNs. We used 92,322 chemicals annotated with LD50 values, SMILES, and other physicochemical properties. The structconvert utility in Schrodinger software (version 2021) was used to convert the SMILES of the 92,322 chemicals to 2D structures in .sdf format. The chemicals were filtered further by removing metal-containing compounds and obtained a final set of 59,373 chemicals. Then, 3D minimization application in Schrodinger’s Canvas module was used to convert the 2D structures to a .sdf file containing 3D structures. We calculated 51 physicochemical descriptors for 59,373 chemicals using the QikProp application in the Schrodinger computational chemistry software https://www.schrodinger.com (accessed on 15 February 2020).

Dataset 1(a) IP/IV/Subcutaneous/Oral—All animals: Out of 59,373 chemicals, 55,856 were annotated with LD50 values and the different routes of animal exposure, such as IP, IV, subcutaneous, and oral. This dataset was further annotated with 26,923 chemicals as nontoxic, and 28,933 were toxic based on the toxic-nontoxic cutoff of LD50 of 500 mg/kg. Randomly selected 5000 chemicals were used as a test set, while the remaining chemicals were used as a training set during each prediction.

Dataset 1(b) Oral—Rat/Mouse: Out of 59,373 chemicals, 22,808 with LD50 were obtained by filtering rat and mouse species via the oral route of exposure. We calculated 31 ADMET (absorption, distribution, metabolism, excretion, and toxicity) properties for the 22,792 chemicals using the ADMETlab platform [19]. Additionally, 12 physicochemical properties, 224 topological properties, and 155 MACCS fingerprints were calculated using the Canvas application of the Schrodinger software. In total, 318 descriptors and 155 fingerprints were calculated for 22,792 chemicals. We then annotated 16,311 chemicals as nontoxic and 6481 as toxic. Randomly selected 4500 chemicals were used as a test set, while the remaining chemicals were used as a training set for each prediction. We used the Toxin and Toxin Target Database (T3DB) 636 rat and oral mouse dataset as an external test set to validate the model developed for the ChemIDplus dataset.

##### National Toxicology Program Data

We used the predictive toxicity models project dataset provided by the National Toxicology Program [20] as an external validation set. This dataset consisted of LD50 values for rat acute oral toxicity and was classified as toxic if LD50 was >500 mg/kg. We calculated 51 property descriptors using the Schrodinger QikProp module.

2a. NTP data as an external validation set for ChemIDplus Oral Rat/Mouse data

The same chemicals in NTP data that also existed in the Oral dataset from ChemIDplus (described in Section 1b) were removed. The final list of 1703 chemicals was obtained as an external validation set. The 1703 chemicals were used as an external validation set for the models developed with oral ChemIDplus training dataset with 51 QikProp descriptors.

2b. NTP data as an external validation set for ChemIDplus IP/IV/Sub/Oral data

The same chemicals in NTP data that also existed in the IP/IV/Sub/Oral dataset from ChemIDplus (described in Section 1a) were removed. The final list of 1648 chemicals was obtained as an external validation set. The 1648 chemicals were used as the external validation set for the models developed with IP/IV/Sub/Oral ChemIDplus training dataset with 51 QikProp descriptors.

##### Toxin and Toxin Target Database (T3DB) Dataset

The 3673 chemicals with toxin data were curated from the T3DB database [21]. Out of 3673 chemicals, we computed 62 descriptors for 778 (Supplementary Table S1) and separated the dataset into the different routes of exposure as below.

3a. Oral exposure data: 687 chemicals with 62 descriptors were separated as Oral data.

3b. IP/IV/Sub/Oral exposure data: 752 chemicals with 62 descriptors were separated as IP/IV/Subcutaneous/Oral data.

#### 2.1.2. Multiclass Classification Models

We classified chemicals with different dose ranges for the development of categorical toxicity models. We classified 687 Oral data (obtained from Section 3a) into four categories: (a) LD50 < 50 mg/kg, (b) 50 mg/kg ≤ LD50 < 500 mg/kg, (c) 500 ≤ LD50 < 1000, and (d) LD50 ≥ 100.

a. T3DB data as an external validation set for ChemIDplus Oral Rat/Mouse data

The same chemicals in T3DB data (Section 3a) that also existed in the oral dataset from ChemIDplus (Section 1b) were removed. The final list of 636 chemicals was retained as the external validation set. Datasets were annotated with toxicity classification at various LD50 cutoffs at 250 mg/kg, 500 mg/kg, 750 mg/kg, and 1000 mg/kg. In total, 318 descriptors and 155 fingerprints (as described for a dataset in Section 1b) were calculated. These data were used as an external validation set for the models developed with the ChemIDplus oral training dataset, as described in Section 1b.

b. EPA data as an external validation set for ChemIDplus Oral Rat/Mouse data

The animal toxicity data of 980 chemicals were retrieved from the EPA. The lowest effect level (lel) chemical dose was considered to determine the toxicity. Various LD50 thresholds considered were 250 mg/kg, 500 mg/kg, 750 mg/kg, and 1000 mg/kg.

#### 2.1.3. Tox21 Challenge Dataset

Tox21 Challenge dataset of 12 different assays and their corresponding 801 descriptors were obtained from the online resource (http://bioinf.jku.at/research/DeepTox/tox21.html accessed on 14 November 2019) [13].

### 2.2. SMILES Preprocessing

SMILES was used as one of the key chemical attributes in our hybrid deep learning model. Raw texts cannot be directly used as input for the deep learning models but should be encoded as numbers. The entire list of SMILES strings was first fit onto the tokenizer to create a dictionary of the set of all the possible characters in the SMILES string and its corresponding index. We assumed that a dictionary D is created where D = {‘C’: 1, ‘=‘: 2, ‘(‘: 3, ‘)’: 4, ‘#’: 5, ‘N’: 6, …, ‘ ‘: M}. This results in every character in the SMILES string being assigned a unique integer value which is the index of the character in the dictionary. Here, M is the total number of unique characters in the SMILES of our dataset. The SMILES entry for every chemical is then converted to a one-hot encoded 2D matrix. One-hot encoding results in each character of the SMILES string to be represented by M bits and the bit at a particular position assigned to that character by the dictionary is set to 1 whereas remaining bits remain as 0 for that character. As an example, acrylonitrile-d3 with SMILES string *C*=*CC*#*N* is one-hot encoded as:

$$\left[\begin{array}{c} C\\ =\\ C\\ C\\ \#\\ N \end{array}\right]=\left[\begin{array}{cccccccc} 1 & 0 & 0 & 0 & 0 & 0 & & 0\\ 0 & 1 & 0 & 0 & 0 & 0 & & 0\\ 1 & 0 & 0 & 0 & 0 & 0 & & 0\\ 1 & 0 & 0 & 0 & 0 & 0 & \cdots & 0\\ 0 & 0 & 0 & 0 & 1 & 0 & & 0\\ 0 & 0 & 0 & 0 & 0 & 1 & & 0\\ & & & \vdots & & & \ddots & \vdots \\ 0 & 0 & 0 & 0 & 0 & 0 & \cdots & 0 \end{array}\right]$$

Thus, as mapped by the dictionary, 1st bit is set to 1 for “C”, 2nd bit is set to 1 for “=”, 5th bit is set to 1 for “#”, and 6th bit is set to 1 for “N”.

A 3D matrix of size K × L × M is obtained eventually, where K is the number of chemicals, L is the maximum length of the SMILES string, and M is the number of all possible characters in the SMILES string from K chemicals (number of entries in the created dictionary). One-hot encoding means converting the integer value of each character in the SMILES to its equivalent binary vector of length M.

### 2.3. The Hybrid Neural Network Model

**Figure 1:**
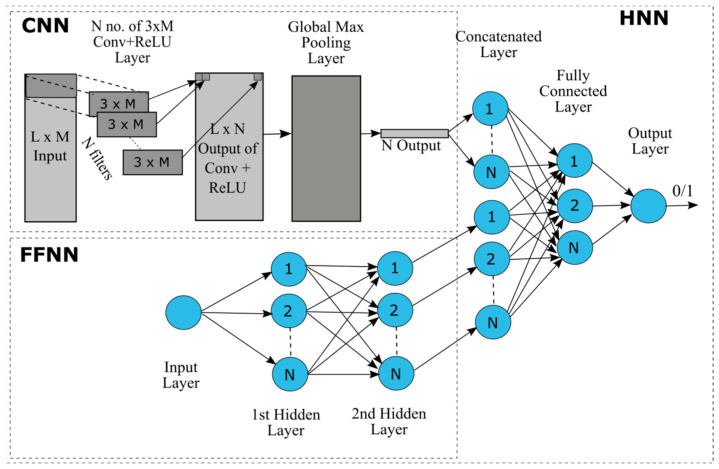
Schematic diagram of the Hybrid Neural Network (HNN) consisting of Convolutional Neural Network (CNN) and Feed-Forward Neural Network (FFNN). L, length of the SMILES string; M; N, number of filters (possibly different at each layer).

The HNN-Tox model is developed in python using the Keras API with Tensorflow in the backend. The model consists of a Convolutional Neural Network (CNN) for deep learning based on structure attributes (SMILES) and a multilayer perceptron (MLP)-type feed-forward neural network (FFNN) for learning based on the remaining features of the chemicals (Figure 1). The basic layers of a CNN include convolutional layer, a non-linearity layer, a pooling or sub-sampling layer, and a fully connected layer. The convolution layer learns and extracts features from the input array, the computing dot product between the weights and a small region of the input matrix. The weights are represented by a matrix, called kernel or filter, smaller in size than the input matrix. To represent the real-world data, the non-linearity layer applies one of the various available activation functions, such as Rectified Linear Unit (ReLU), sigmoid, and tanh to introduce non-linearity in the model. The activation function ReLU represented mathematically as max (0, x), is used in the model that replaces all the negative values with zeros. The derivative of ReLU is always 1 for positive input, which counteracts the vanishing gradient problem during the backpropagation. Pooling or sub-sampling reduces the dimension of the data while retaining the important information in the data. Max pooling is used in the model for subsampling. The fully connected layer performs the final classification implementing the softmax activation function in the case of multiclass classification and the sigmoid activation function in the case of binary classification. In a fully connected layer, every neuron in the current layer is connected to every neuron in the previous layer. A multilayer perceptron (MLP)-type feed-forward neural network (FFNN) contains one or more hidden layers. The 3D array of one-hot encoded SMILES strings was the input for the CNN and chemical descriptors were the input for the FFNN. The output pooling layer of the CNN was merged with the final fully connected layer of FFNN to perform the classification task.

### 2.4. Parameter Tuning

We implemented hyperparameter tuning to improve the performance of the model. A Hyperopt package from python that uses Tree-structured Parzen Estimator (TPE) method was used for hyperparameter optimization. This approach requires defining the objective function that the fmin () function minimizes, the parameter space over which the search is performed, and the number of experiments to run. The Area Under the receiver operating characteristic Curve (AUC) was the metric used to evaluate each model’s performance. Class imbalance is a common problem while modeling toxicity data and AUC is a better metric to optimize than accuracy.

### 2.5. Other Machine-Learning Algorithms

To test HNN-Tox performance, we developed several other models based on other machine-learning algorithms, such as Random Forest and Bootstrap Aggregation (Bagging), Bagged Decision Tree, and Adaptive Boosting (AdaBoost).

### 2.6. Ensemble Model

An ensemble of model predictions is considered an excellent option to optimize the model’s performance. To boost overall performance, we made ensemble predictions using the Random Forest, Bagging, and Adaboost methods. The ensemble method derived by Mayr et al. [13] for calculating ensemble probabilities was used to make the ensemble model’s final prediction [Supplementary Equation (S1)].

### 2.7. Model Performance Evaluation

All the results presented are the average of 10 simulation run repeats for the ChemIDplus datasets and 30 simulation repeats for the T3DB and Tox21 Challenge datasets. Thus, 20% of the dataset was randomly separated as the test set; the remaining data were used as the training set, which is similar to 10-fold cross-validation except that the test sets were randomly selected each time. The performance of each model was evaluated based on the accuracy and the Area Under the receiver operating characteristic Curve (AUC). The AUC gives the probability of a positive outcome being ranked before the negative outcome and is a better metric for evaluating a binary classifier than accuracy [22,23]. The models were also assessed for their sensitivity, specificity, and precision (Supplementary Equation (S2)). Other models were used for comparisons and for ensemble predictions to improve the overall performance of the HNN-Tox.

## 3. Results and Discussion

**Figure 2:**
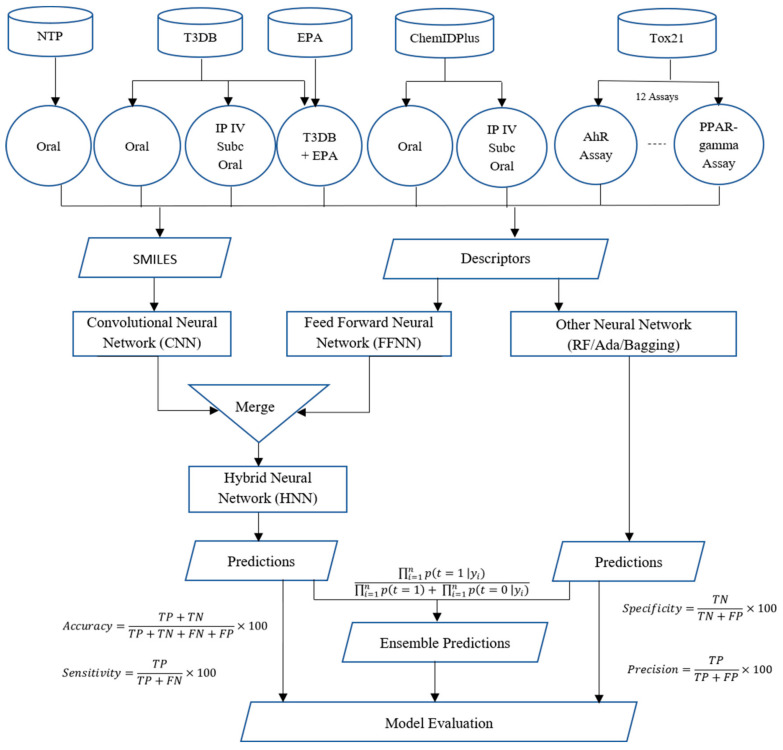
The overall flowchart of the HNN-Tox toxicity prediction processes with detailed data preparation for each data source is indicated (see Methods section for details).

In this study, we present a deep-learning-based novel hybrid neural network (HNN) method called HNN-Tox that consists of a CNN and FFNN framework to predict chemical toxicity at different doses and dose ranges. The overall workflow of the HNN-Tox process for chemical toxicity prediction is shown in Figure 2. We collected and used the largest dataset ever reported in the literature. Such a large data size is proven to help improve accuracy in many machine-learning scenarios, such as overfitting models with high variance [24]. As detailed in the Methods section, thousands of chemicals annotated with experimental toxicity data were downloaded to begin the toxicity prediction process. The data were then processed to separate them based on their route of exposure with different toxic dose LD50 cutoff values. Next, molecular descriptors and SMILES strings were computed. All models were developed with a large chemical domain coverage with various data sizes (balanced and imbalanced). The input training dataset with varying size is prepared with a maximum dataset containing 59,373 chemicals. The test sets were compiled from the internal and external validation datasets. We classified the chemicals into different categories based on their toxicity level and the results are presented in the following section. The binary and multiple classification models were developed to make toxicity predictions at different dose cutoffs. We first developed binary classification models to predict toxic or nontoxic chemicals. Next, multiclass classification models were developed to predict chemicals’ degree of toxicity (categorical). The classification models were developed using HNN-Tox and other machine-learning algorithms, including Random Forest (RF), Bootstrap Aggregation (Bagging), and Adaptive Boosting (AdaBoost). We used various training dataset sizes, dose, routes of exposure, descriptors, and fingerprints to test the dependency and performance variations in the neural network models. The class imbalance in the prediction capability of the deep learning model was also studied. Finally, the performance of the HNN-Tox was computed and compared with other machine-learning algorithms, including Random Forest (RF), Bootstrap Aggregation (Bagging), and Adaptive Boosting (AdaBoost).

### 3.1. Toxicity Prediction Using Binary Classification

#### 3.1.1. ChemIDplus Data Predictive Toxicity Analysis (Oral Toxicity: Rat and Mouse)

**Figure 3:**
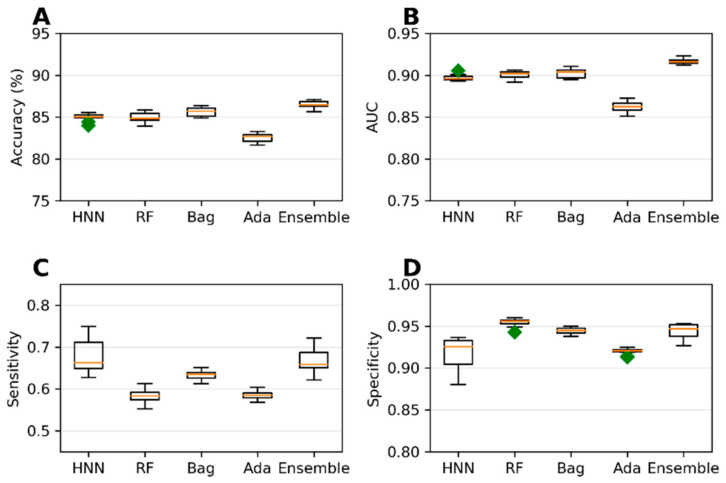
(**A**) Accuracy, (**B**) AUC, (**C**) sensitivity, and (**D**) specificity for the ChemIDplus oral data as predicted by HNN, RF, Bagging, AdaBoost, and the Ensemble methods with additional descriptors from ADMETlab and Canvas.

The collected 386,620 chemicals from the ChemIDPlus online database, annotated with experimental LD50 data, are multispecies data. We separated the data annotated with oral rat and mouse acute toxicity (LD50) and obtained 22,792 chemicals. The 318 descriptors and 155 MACCS fingerprints were computed for 22,792 chemicals. The predictive models were developed with an LD50 threshold value of 500 mg/kg. For the HNN-Tox, the FFNN was developed based on the 318 descriptors and two CNNs were developed based on the 155 fingerprints and SMILES string separately. Independently, the RF, Bag, and Ada models were developed based on the 318 descriptors. The HNN-Tox, RF, and Bag models exhibit similar accuracy and AUC (Figure 3A,B). In contrast, the HNN-Tox model’s sensitivity to correctly identify the positives, i.e., the toxic chemicals, was significantly higher than the other models (Figure 3C). The ensemble model (combination of RF, Bag, and Ada) improved the performance by providing high accuracy of 86.50% and an AUC of 91.65%. Overall, HNN-Tox performance was optimal for all the statistical metrics compared to the other models, which fluctuate among different metrics. The prediction accuracy achieved with a qualitative binary toxicity prediction model by Sharma et al. [25] was 93%. In their model, the training set consisted of chemicals obtained from the T3DB database as a positive dataset and human metabolites as a negative dataset. Evidently, such high accuracy is possible because the compound source is different for the toxic and nontoxic groups. Further, compounds in the T3DB database were compositionally distinct from the metabolites, as revealed by their compositional analysis. In contrast, in our dataset, the toxic and nontoxic compounds were obtained from the same source and represent real-life chemicals. Thus, our model is more generalized, with diverse chemicals in both toxic and nontoxic groups.

Next, to investigate the effect of descriptors and SMILES on the predictive performance, we developed models with 51 Schrodinger QikProp descriptors (instead of 318 descriptors) in addition to the SMILES for the CNN (without MACCS fingerprints). Notably, in the absence of many descriptors, such as ADMET and fingerprints, the accuracy and AUC of RF, Bagging, and Ada are decreased significantly (Table 1). This means that the absence of 277 descriptors plays a significant role in the prediction performance. At the same time, the HNN-Tox was not affected and maintained similar accuracy and AUC (Table 1). This is because the HNN-Tox uses the additional SMILES as input features that enriched the model to learn based on the structure of the compound and enabled the model to compensate for the missing descriptors in the toxicity prediction.

##### External Validation of the ChemIDplus Oral Data Model Predictions

To ensure the prediction accuracy of the models developed for the ChemIDplus dataset, the performance was evaluated by making predictions for external datasets that were not used to develop the models. The T3DB and NTP datasets (see Methods section) were used as external validation.

A.T3DB data as an external validation set

We first removed the common chemicals present in both T3DB and ChemIDplus datasets from the ChemIDplus training dataset. The total chemicals in the training set then included 22,438 rat and mouse oral toxicity data and the test set included 636. The models predicted an accuracy of 76.94%, 75.69%, 72.76%, 74.37%, and 75.28% and an AUC of 0.83, 0.81, 0.79, 0.80, and 0.84 for the HNN-Tox, RF, Bag, Ada, and Ensemble, respectively (Supplementary Figure S1). These results showed that the models could make predictions for the external dataset with high accuracy and AUC, proving their predictive ability. The HNN-Tox displayed the best performance for the statistical predictor accuracy, and AUC, with sensitivity and precision, was even significantly higher than the other models, proving the model’s ability to identify toxic chemicals correctly (Supplementary Figure S1).

B.NTP data as an external validation set

Next, we used the NTP dataset as the second external validation dataset with rat and mice oral toxicity. Models were developed with 1703 chemicals and 51 QikProp descriptors (Section 2a of the Methods section). The models predicted accuracy of 73.29%, 75.23%, 75.39%, 69.82%, and 75.44% for the HNN-Tox, RF, Bagging, AdaBoost, and Ensemble, respectively (Supplementary Figure S2). The AUCs of the models were 0.77, 0.78, 0.77, 0.70, and 0.78. All models performed equally well across the NTP validation dataset. The training set is a mix of the rat and mice data, but the validation set comprises only the rat data. Thus, the training set is not very specific to rat LD50 values, which could be the reason for the decrease in the performance of the models.

#### 3.1.2. ChemIDplus Data Predictive Toxicity Analysis (IP/IV/Subcutaneous/Oral Toxicity: All Animals/Birds)

**Figure 4:**
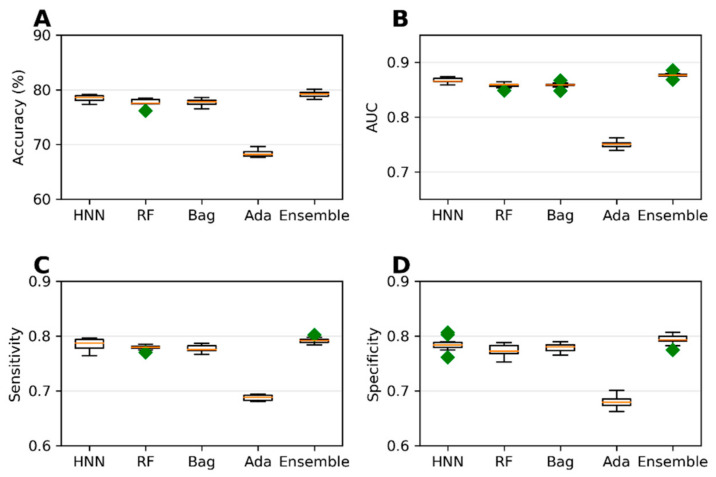
(**A**) Accuracy, (**B**) AUC, (**C**) sensitivity, and (**D**) specificity for the ChemIDplus IP/IV/Sub/Oral data as given by HNN, RF, Bagging, AdaBoost, and the Ensemble methods.

Extending the domain coverage of the data enhances the model’s applicability. Thus, we sought to apply our models to a more generalized larger dataset that consists of 55,856 chemicals from the ChemIDplus data. These chemicals were annotated with the experimental LD50 of the animals and birds via the IP/IV/Subcutaneous/Oral route of administration. Molecular descriptors (51) were computed using the Schrodinger QikProp tool. The model’s predictive performance developed on this more generalized large dataset compared to the models developed on smaller rat and mice oral data; the accuracy decreased from 84.96% (Figure 3) to 78.43% (Figure 4) for the HNN-Tox. At the same time, the decrease was minimal for AUC (from 0.88 to 0.86). This means the model maintains its performance and can distinguish chemicals into toxic and nontoxic classes. The HNN-Tox model’s sensitivity was increased from 0.67 to 0.78 (Figure 4C), whereas specificity decreased from 0.92 to 0.78 (Figure 4D). Overall, the HNN-Tox model performance was optimal compared to the other machine-learning models (Figure 4).

##### Validation of the ChemIDPlus IP/IV/Sub/Oral Data Models

We next evaluated the prediction capability of the models for this more generalized larger set (55,856 chemicals). The NTP data were used as an external validation dataset that contains 1648 chemicals with 51 descriptors (Section 2b of the Methods section). For this NTP external validation set, the accuracy was 60% for HNN-Tox, with much less accuracy predicted by other models (Supplementary Figure S3A). The AUC was around 0.72 for all the models (Supplementary Figure S3B). The other statistical metrics are also lower in this external validation data (Supplementary Figure S3B,C). The moderate prediction performance of the models was obtained for this NTP external validation set when compared to the internal test set. This is because the internal training and test sets comprise all species with homogenous data. In contrast, the NTP external validation set contains rat-only acute toxicity LD50 data, mostly heterogeneous data.

#### 3.1.3. T3DB the Oral Route of Exposure Dataset Predictive Toxicity Analysis

**Figure 5:**
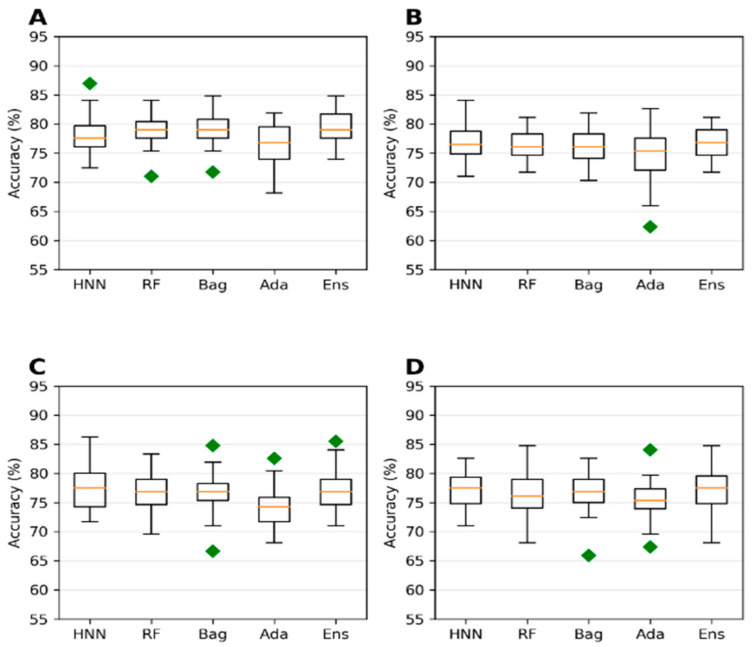
Predicted accuracy of T3DB data obtained via oral route of exposure with cutoffs at (**A**) 250 mg/kg, (**B**) 500 mg/kg, (**C**) 750 mg/kg, and (**D**) 1000 mg/kg.

**Figure 6:**
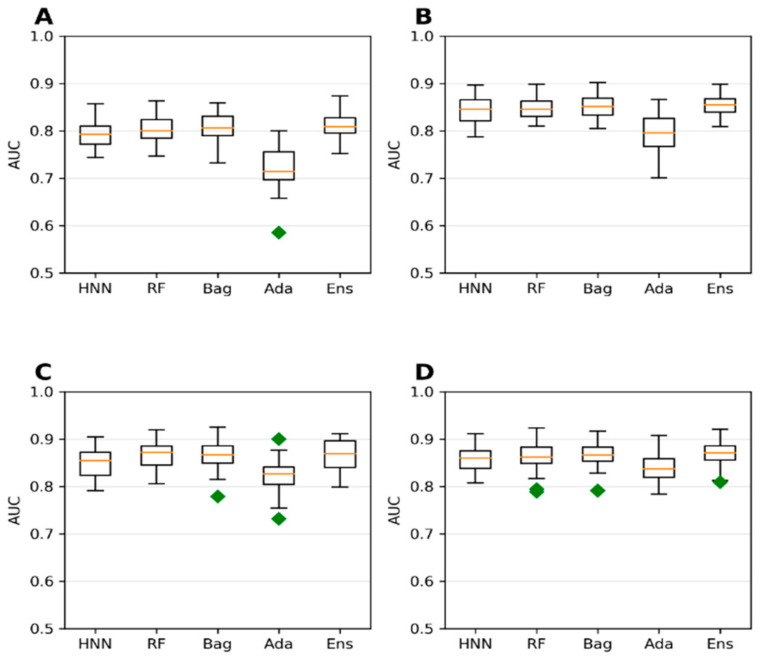
The AUC for toxins LD50 data obtained via oral route of exposure with various cutoffs at (**A**) 250 mg/kg, (**B**) 500 mg/kg, (**C**) 750 mg/kg, and (**D**) 1000 mg/kg by HNN, RF, Bagging, AdaBoost, and the Ensemble methods.

The T3DB oral route of exposure data of 687 chemicals with 62 descriptors was used here (Section 3a in the Methods section). Models were developed using four different LD50 cutoffs: 250 mg/kg, 500 mg/kg, 750 mg/kg, and 1000 mg/kg. The accuracy is highest when the LD50 threshold is set at 250 mg/kg, whereas the AUC was lowest in this category (Figure 5A and Figure 6A). The model lost its ability to accurately rank toxic substances over nontoxic ones, resulting in lower AUC at the 250 mg/kg threshold. The lower AUC is due to a higher imbalance in data (ratio of 3.4:1 for nontoxic:toxic) when the LD50 threshold is 250 mg/kg compared to those obtained for the LD50 threshold >250 mg/kg (ratio of 1.41:1 to 2:05 for nontoxic:toxic) (Figure 5). An imbalanced dataset was included, with most samples being classified into one class, while very few were classified into the other class. Such skewed imbalanced data scenarios in the test set result in apparently very high accuracy. The model trained on such a dataset predicts the test samples as belonging to the majority class, often ignoring the minority class. These results suggest that the model with training and test datasets that is more skewed towards one class may predict with high accuracy, whereas the AUC of the model tends to be low.

#### 3.1.4. T3DB Dataset Predictive Toxicity Analysis via IP/IV/Subcutaneous Oral Route of Exposure

To study the effect on the toxicity prediction for the chemicals administered via various routes of exposure, the toxin data from the T3DB were separated to include intraperitoneal (IP), intravenous (IV), subcutaneous oral routes of exposure (IPIVSubOral) data (Supplementary Table S2). The models were developed on this combined route of exposure data, including 752 chemicals computed with 62 descriptors (Section 3b in Materials and Methods). Notably, the change in the toxicity determination method based on the route of chemical administration changed the overall toxic:nontoxic ratio for the four LD50 threshold values (Supplementary Figures S4 and S5). The category with 1000 mg/kg threshold has the most imbalanced data, with a ratio of 3.2:1. The accuracy is highest with 78.96%, 80.38%, 79.73%, 74.71%, and 80.64% for HNN, RF, Bagging, Ada, and Ensemble methods, respectively, for the 1000 mg/kg category (Supplementary Figure S4D). In contrast, the AUCs are lower for the categories with 1000 mg/kg and 250 mg/kg thresholds (Supplementary Figure S5A,D). At 750 mg/kg threshold with more balanced data, the AUC is higher with 0.85, 0.85, 0.85, 0.79, and 0.86 for HNN-Tox, RF, Bagging, Ada, and Ensemble (Supplementary Figure S5C), whereas at 500 mg/kg threshold, the AUC ~0.86 for all the models (Supplementary Figure S5B). Taken together, more imbalanced data negatively affect the model classification performance with lower AUC. There is no significant change in accuracy or AUC of the models developed with IP, IV, and subcutaneous routes of chemical administration when compared to the models developed for the oral route of exposure (Supplementary Figures S4 and S5). In contrast, the accuracy and AUC were changed in both cases’ toxic to nontoxic chemical ratio upon comparing the oral route of administration (Figure 5 and Figure 6) and IP/IV/Suboral route of administration (Supplementary Figures S4 and S5). Adding additional data (non-oral) from a different route of exposure did not increase the accuracy or AUC of the model (Supplementary Figure S5). This is because the additional non-oral route of administration data does not contribute sufficient toxicity information to the oral-route training dataset. Further, the model’s performance depends on the quality of the data and the class proportion (i.e., the ratio of the two classes: toxic and nontoxic).

#### 3.1.5. Predictive Toxicity Analysis of Combined T3DB and EPA Dataset

**Figure 7:**
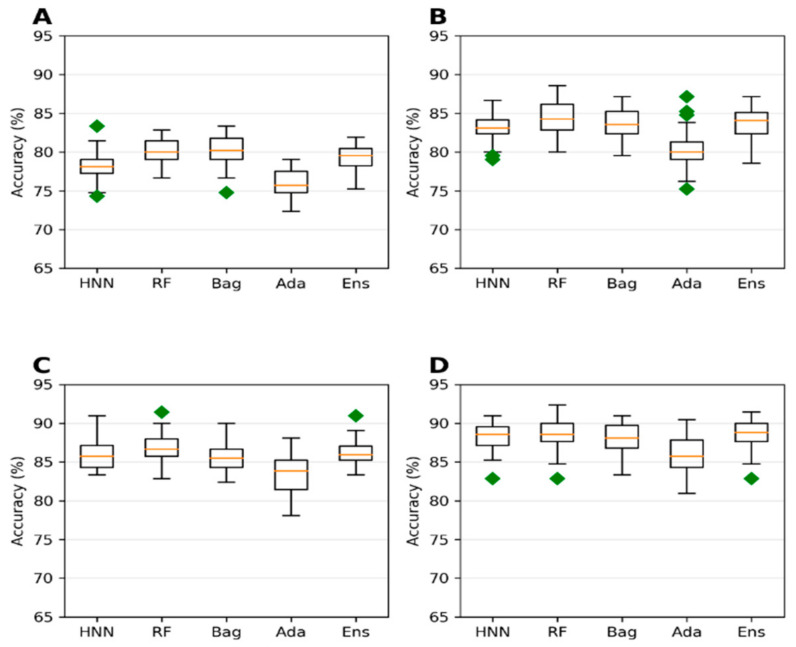
Accuracy for the combined data (toxin data from T3DB + animal toxicity data from EPA) with cutoffs at (**A**) 250 mg/kg, (**B**) 500 mg/kg, (**C**) 750 mg/kg, and (**D**) 1000 mg/kg by HNN, RF, Bagging, AdaBoost, and the Ensemble methods.

**Figure 8:**
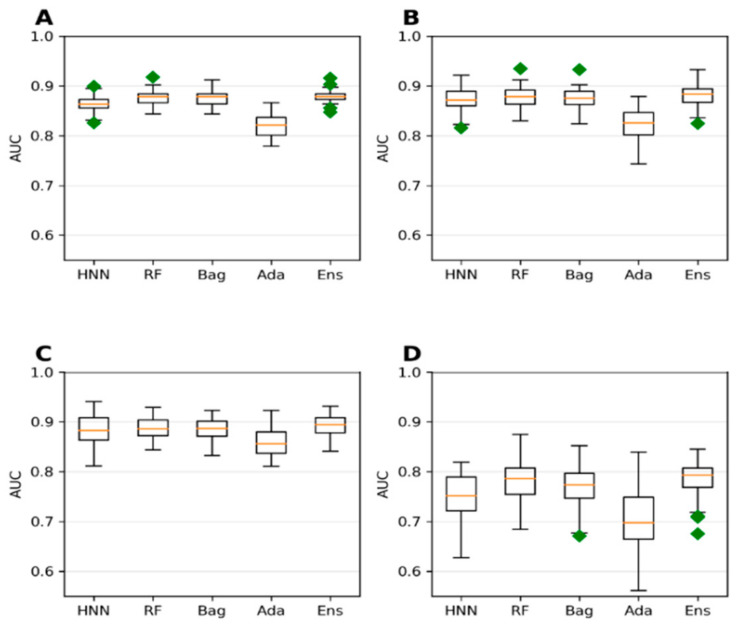
AUC for the combined data (toxin data from T3DB + animal toxicity data from EPA) with cutoffs at (**A**) 250 mg/kg, (**B**) 500 mg/kg, (**C**) 750 mg/kg, and (D) 1000 mg/kg by HNN, RF, Bagging, AdaBoost, and the Ensemble methods.

Next, we investigated if achieving a larger dataset by combining chemicals from different sources and toxicity affects the model’s predictive ability. The animal LD50 data from the T3DB and the EPA data with lel dose values were combined to form a single dataset (Supplementary Table S4). Both datasets were not separated based on the route of administration. The 778 chemicals from T3DB were combined with 427 chemicals from EPA to obtain 1054 unique chemicals. The model from this combined dataset exhibited the highest accuracy when the threshold value was set at 1000 mg/kg (Figure 7D). This is a trend towards similar accuracy of 88.16%, 88.41%, 88.02%, 85.95%, and 88.51% for the HNN-Tox, RF, Bagging, AdaBoost, and ensemble methods, respectively (Figure 7D). In contrast, the AUC for the same dataset was lower with 0.75, 0.78, 0.77, 0.71, and 0.78, respectively (Figure 8D). This combined T3DB + EPA dataset achieved a very high accuracy of greater than 88% but with lower AUC. This is because the combined data are highly imbalanced, with a ratio of 8.32 to 1 for the 1000 mg/kg threshold.

Further, the 250 mg/kg threshold accuracy was the lowest compared to the 500 mg/kg and 750 mg/kg thresholds (Figure 7A,C). In contrast, the AUC 0.8 was high for the same thresholds for which the dataset ratio toxic:nontoxic varied from 1.44 to 2.15 (Figure 8A–C). The dataset with a 750 mg/kg threshold (nontoxic:toxic data ratio of 2.15:1) yielded the highest AUC (approximately 0.88) (Figure 8C), whereas the accuracy was ~86% (Figure 7C). These predictive trends of the models showed that optimal AUC could be achieved if the class is balanced (ratio between 1 and 2) for smaller datasets. Highly imbalanced data apparently resulted in overfitting that increased the accuracy. Still, their corresponding robust AUC shows the model’s ability to correctly rank the toxic over the nontoxic substances (Figure 8).

Considering the dataset with the highest AUC, i.e., 1000 mg/kg threshold T3DB Oral data (Figure 6D) and 750 mg/kg threshold T3DB IP/IV/SubOral data (Supplementary Figure S5C), their highest AUCs are 0.858 and 0.848 with an accuracy of 77% (Figure 5D and Supplementary Figure S4C). Increasing the data size by adding additional animal toxicity data from EPA increased the model’s accuracy significantly from 77% to 86% (Figure 7C). The AUC was also increased to 0.88 (Figure 8C). The highest accuracy achieved was 88% with the highly imbalanced dataset for the 1000 mg/kg threshold (Figure 7D) due to overfitting; however, the AUC was significantly reduced to 0.75 (Figure 8D). If the dataset was not highly imbalanced, augmenting the training set with additional data can increase the accuracy while maintaining the AUC.

#### 3.1.6. Predictive Toxicity Analysis of the Tox21 Challenge Dataset

Next, we sought to assess how well HNN-Tox can predict compounds’ interference in biochemical pathways, an entirely unique and different dataset when compared to those used in the previous sections. The Tox21 Challenge data contains 12 different experimental assay data from the National Institute of Health database. These data were generated from nuclear receptor signaling and stress pathway assays run against Tox21’s 10,000-compound library (Tox21 10K). We obtained 801 molecular descriptors computed for this dataset from Mayr et al. [13]. Then, the models were developed to predict whether binary endpoints are active or not active. The overall performance of all models was optimal, as seen in Table 2. The accuracy and AUC of the HNN-Tox were similar and sometimes better than the other methods (Table 2). The AUC of the HNN-Tox was fairly comparable to the DeepTox [13] model (the Tox21 challenge winning model) AUC in many assays, despite not increasing the performance of the models with additional data (Table 2). Overall, the ensemble method improved the accuracy and the AUC for most assay data.

### 3.2. Toxicity Prediction Using Multiclassification

#### Predictive Categorical Toxicity Analysis of the T3DB Oral Data

**Figure 9:**
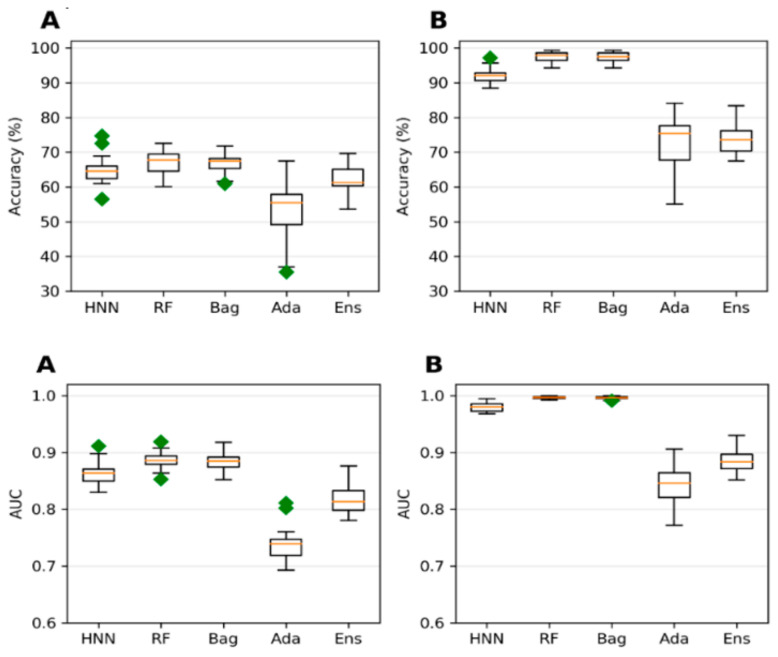
Accuracy and AUC for the multiclass classification (**A**) toxins Oral data and (**B**) toxins oral data + Oversampling by HNN, RF, Bagging, AdaBoost, and the Ensemble method.

The degree of toxicity (categorical toxicity) at a different dose range is important in the field of toxicology. The degree of toxicity can vary from substance to substance. Therefore, assessing the hazardous substances’ categorical toxicity is imperative. These substances can be categorized based on their toxicity severity level. To predict the categorical toxicity of substances, the toxins from the T3DB database were classified into four categories: (a) LD50 < 50 mg/kg, (b) 50 mg/kg ≤ LD50 < 500 mg/kg, (c) 500 ≤ LD50 < 1000, and (d) LD50 ≥ 1000 (Supplementary Table S5). The multiclass classification models based on the HNN-Tox, RF, Bagging, and SVM algorithms were developed for this categorized toxin dataset. The accuracy is moderate for all the models (Figure 9A), whereas the corresponding micro AUC is higher, as shown in Figure 9A. We used micro averaging because in multiclass classification with the imbalanced dataset, micro averaging of any metric is preferred compared to macro averaging. Micro averaging involves calculating the AUC by converting the data in multiple classes into binary classes. In contrast, macro averaging involves averaging the AUC obtained from each class by giving them equal weight. Giving equal weights to each class while calculating the average produces results biased toward the majority class. Class imbalance is one of the major problems encountered with toxicity data. This problem is more apparent in the case of multiclass classification, as separating chemicals into multiple categories increases the possibility of highly imbalanced classes with an insufficient number of samples in some classes for training purposes. Hence, a decrease in the accuracy of the model developed based on T3DB oral data in the multiclass classification may be due to these minority classes with a smaller training set (Figure 9A). The random oversampling method, a simple and competitive method compared with other complex oversampling methods [26,27], was applied to overcome the model’s poor performance caused by an imbalanced dataset (Figure 9B). One hundred samples were randomly selected as a test set. The remaining chemicals in classes 1, 2, and 3 were duplicated to increase the number of samples in the raining set to 100, 200, and 100, respectively, drawing them randomly with replacement. By sampling with a replacement for multiclass classification, the model accuracy and AUC improved significantly (Figure 9B). Taken together, oversampling that results in more data to train in each multiclass enhances the performance of the models.

## 4. Conclusions

We developed a hybrid neural network framework method called HNN-Tox to predict dose-range chemical toxicity. The performance of the HNN-Tox was compared with other machine-learning methods. The optimal performance of the HNN-Tox is evident in most cases. We also analyzed the HNN-Tox performance on the learning feature dependency, feature variations, route of administration, dose, and dataset size. The results suggest that the machine-learning methods depend on these variations and the imbalanced dataset. The HNN-Tox performed well, even after decreasing the number of chemical descriptors to train. For example, compared to the other methods, the HNN-Tox maintained similar accuracy and AUC when the chemical descriptors were decreased from 318 to 51 for the ChemIDplus data. Notably, adding the SMILES feature with the descriptors enriched the HNN-Tox model to learn based on the structure of a chemical and compensate for the missing descriptors. Further, the HNN-Tox demonstrates overall optimal toxicity-predicting capability with the external validation chemical sets.

The binary classification models developed on a smaller dataset showed the impact of class imbalance. Accuracy is increased and the AUC is decreased with the increase in class imbalance. For example, the highest accuracy was achieved in the case of the combined dataset (EPA + T3DB) when the class imbalance ratio was 8.32:1 for the LD50 cutoff 1000 mg/kg; however, the AUC was lowest in this model. These models showed that optimal AUC could be achieved if the class is balanced (ratio between 1 and 2) for the smaller datasets. When the training set is enriched with additional data with highly balanced data, the accuracy increases without affecting the AUC. This means that the high accuracy achieved in the case of imbalanced data was due to overfitting.

The models developed for the multiclass categorical classifications predicted chemical toxicity with moderate accuracy and higher AUC. The decrease in accuracy is due to the class imbalance, which is more evident in the case of multiclass classification, as separating chemicals into multiple categories increases the possibility of highly imbalanced classes, with an insufficient number of samples in some classes for training purposes. A random oversampling method was used to augment the model performance by counteracting the data imbalance, significantly improving the accuracy and the AUC. Additionally, the HNN-Tox performed optimally in predicting active and inactive to entirely different datasets obtained from the EPA Tox-21 data challenge containing 12 different target-based pathways’ cell assay data.

In summary, in a large-scale prediction of dose-dependent and categorical toxicity, with different dataset sizes and varying features, the HNN-Tox method performed optimally well most of the time without needing many descriptors. Further, the HNN-Tox is not sensitive when applied to diverse chemicals and maintains optimal performance. Consequently, the HNN-Tox can be used to predict the toxicity of any chemical type with a broad applicability domain. Additionally, the HNN-Tox could act as a new approach method (NAM) to help prioritize the chemicals and avoid conducting a costlier and more time-consuming animal experiment to identify toxic chemicals. Due to the hybrid nature of the HNN-Tox method, it has broad applicability in predicting toxicity and carcinogenicity of chemical mixtures and those studies are in progress.

## 5. Limitations

The HNN-Tox, in its current form, has some limitations that are reflected in the lower performance in some cases. We used a random split in some datasets to split the data into training and test data. This may sometimes lead to overestimating the model’s performance because similar molecules could be in the training and the test set. We used various in vitro and in vivo datasets, multispecies, and various dataset sizes with varying features. Obviously, these data are very complex and require a significant optimization routine to obtain an excellent performance overall. Therefore, in many ways, we will improve the HNN-Tox method. We will introduce multi-task learning, as deep neural networks support multi-task learning, which generally boosts performance. We will add a fine-tuned, Fully Convolutional Neural Network (FCNN) framework. We will introduce more hyper-optimization parameters. We will introduce several optimization routines and extend the simulation run to 50. In the next routine, nested cross-validation will also be used for model comparison to give all approaches a chance to find a good hyperparameter. This would enable us to measure the performance of the methods without bias. We will use cluster-based nested cross-validation (i.e., cluster cross-validation), which is based on molecular similarities.

The Dataset 1(a) IP/IV/Subcutaneous/Oral—All animals, Dataset 1(b) Oral— Rat/Mouse, 2a NTP data as an external validation set for ChemIDplus Oral Rat/Mouse data. The dataset partition may be illogical because these datasets are from different animal models or different administration methods in the same dataset and may not correspond to the same endpoint. However, in this first version of proof-of-concept studies, we wanted to try the performance of the HNN-Tox method to all types of data partition in the initial phase of this project from small to large, different animal or species data and different administration data. Further, we focused on more generalized predictions. Thus, we made several data partitions starting from mouse only, rat only, mouse/rat, bird data, and other species data individually and combined. Similarly, we tried and made the data partition with appropriate administration data, such as IP, IV, subcutaneous, oral, etc. However, when we used the external validation set, we ensured most of them had no heterogeneous data among training and test datasets. In the next version, we will further fine tune the HNN-Tox to make more appropriate considerations about the dataset partition and focus on more specific examples with specific chemicals and classes, dose, administration, animal/species, and endpoints.

## Figures and Tables

**Table 1:** Accuracy % and AUC for the ChemIDplus oral data with 51 descriptors and 318 descriptors.

	No of Descriptors	HNN-Tox	RF	Bag	Ada
**Accuracy %**	318	84.96	84.84	85.62	82.53
	51	84.11	82.07	82.05	76.24
**AUC**	318	0.897	0.901	0.902	0.862
	51	0.887	0.865	0.861	0.765

**Table 2:** Accuracy and AUC of Tox21 challenge data with 801 descriptors and SMILES for 12 different assays.

		AhR	AR	ARE	AR-LBD	Aromatase	ATAD5	ER	ER-LBD	HSE	MMP	P53	PPAR-Gamma
**Accuracy**	**HNN-Tox**	89.1	97.8	83.2	98.2	92.4	93.7	90.8	96.7	96.6	89.6	93.1	94.7
	**RF**	90.4	97.9	83.5	98.3	92.8	93.9	91.9	97.1	96.5	90.5	93.3	94.7
	**Bag**	89.8	97.9	83.1	98.4	92.8	94.6	91.0	96.4	96.8	91.1	93.3	94.7
	**Ensemble**	90.3	97.9	83.6	98.4	92.8	93.9	91.2	97.1	96.6	90.7	93.4	94.7
**AUC**	**HNN**	0.886	0.783	0.775	0.756	0.769	0.782	0.751	0.741	0.774	0.917	0.828	0.733
	**RF**	0.895	0.718	0.768	0.709	0.772	0.759	0.769	0.757	0.767	0.919	0.784	0.699
	**Bag**	0.897	0.741	0.759	0.718	0.761	0.749	0.751	0.766	0.776	0.908	0.765	0.711
	**Ensemble**	0.905	0.767	0.786	0.720	0.788	0.781	0.767	0.781	0.792	0.928	0.801	0.730
	**DeepTox**	0.928	0.807	0.840	0.879	0.834	0.793	0.810	0.814	0.865	0.942	0.862	0.861

## Data Availability

Not applicable.

## Supplementary Materials

The following are available online at https://www.mdpi.com/article/10.3390/toxics10110706/s1, Figure S1: T3DB external validation dataset performance metrics; Figure S2: NTP dataset performance metrics; Figure S3: NTP external validation dataset performance metrics; Figure S4: Accuracy of the Toxins data with IP, IV, Subcutaneous and Oral route of exposure with various LD50 cutoff; Figure S5. AUC for Toxins data obtained with IP, IV, Subcutaneous and Oral route of exposure with various LD50 cutoff; Table S1: T3DB Toxins data annotated with different LD50 threshold values; Table S2. T3DB Toxins IP/IV/Subcutaneous/Oral route of exposure data annotated with different LD50 threshold values; Table S3. Animal toxicity data from the EPA annotated with different LD50 threshold values; Table S4. Combined data after merging animal toxicity data from the EPA (Table S1) and toxins data from the T3DB (Table S3) annotated with different LD50 threshold values; Table S5. Distribution of Toxins Oral data among four classes in the multiclass classification.

## References

[1] Issa N.T., Wathieu H., Glasgow E., Peran I., Parasido E., Li T., Simbulan-Rosenthal C.M., Rosenthal D., Medvedev A.V., Makarov S.S. (2022). A novel chemo-phenotypic method identifies mixtures of salpn, vitamin D3, and pesticides involved in the development of colorectal and pancreatic cancer. Ecotoxicol. Environ. Saf..

[2] Xu Y., Pei J., Lai L. (2017). Deep Learning Based Regression and Multiclass Models for Acute Oral Toxicity Prediction with Automatic Chemical Feature Extraction. J. Chem. Inf. Model..

[3] Wu K., Wei G.-W. (2018). Quantitative Toxicity Prediction Using Topology Based Multitask Deep Neural Networks. J. Chem. Inf. Model..

[4] Lu J., Peng J., Wang J., Shen Q., Bi Y., Gong L., Zheng M., Luo X., Zhu W., Jiang H. (2014). Estimation of Acute Oral Toxicity in Rat Using Local Lazy Learning. J. Cheminform..

[5] Chavan S., Friedman R., Nicholls I.A. (2015). Acute Toxicity-Supported Chronic Toxicity Prediction: A k-Nearest Neighbor Coupled Read-Across Strategy. Int. J. Mol. Sci..

[6] Cherkasov A. (2005). Inductive QSAR Descriptors. Distinguishing Compounds with Antibacterial Activity by Artificial Neural Networks. Int. J. Mol. Sci..

[7] Zhang L., Ai H., Chen W., Yin Z., Hu H., Zhu J., Zhao J., Zhao Q., Liu H. (2017). CarcinoPred-EL: Novel Models for Predicting the Carcinogenicity of Chemicals Using Molecular Fingerprints and Ensemble Learning Methods. Sci. Rep..

[8] Tanabe K., Lučić B., Amić D., Kurita T., Kaihara M., Onodera N., Suzuki T. (2010). Prediction of Carcinogenicity for Diverse Chemicals Based on Substructure Grouping and SVM Modeling. Mol. Divers..

[9] Li X., Chen L., Cheng F., Wu Z., Bian H., Xu C., Li W., Liu G., Shen X., Tang Y. (2014). In Silico Prediction of Chemical Acute Oral Toxicity Using Multi-Classification Methods. J. Chem. Inf. Model..

[10] Hinton G., Deng L., Yu D., Dahl G.E., Mohamed A., Jaitly N., Senior A., Vanhoucke V., Nguyen P., Sainath T.N. (2012). Deep Neural Networks for Acoustic Modeling in Speech Recognition: The Shared Views of Four Research Groups. IEEE Signal Process. Mag..

[11] Traore B.B., Kamsu-Foguem B., Tangara F. (2018). Deep Convolution Neural Network for Image Recognition. Ecol. Inform..

[12] Lin T., RoyChowdhury A., Maji S. Bilinear CNN Models for Fine-Grained Visual Recognition. Proceedings of the 2015 IEEE International Conference on Computer Vision (ICCV).

[13] Mayr A., Klambauer G., Unterthiner T., Hochreiter S. (2016). DeepTox: Toxicity Prediction Using Deep Learning. Front. Environ. Sci..

[14] Dahl G.E., Jaitly N., Salakhutdinov R. (2014). Multi-Task Neural Networks for QSAR Predictions. arXiv.

[15] Fernandez M., Ban F., Woo G., Hsing M., Yamazaki T., LeBlanc E., Rennie P.S., Welch W.J., Cherkasov A. (2018). Toxic Colors: The Use of Deep Learning for Predicting Toxicity of Compounds Merely from Their Graphic Images. J. Chem. Inf. Model..

[16] Jimenez-Carretero D., Abrishami V., Fernández-de-Manuel L., Palacios I., Quílez-Álvarez A., Díez-Sánchez A., del Pozo M.A., Montoya M.C. (2018). Tox_(R)CNN: Deep Learning-Based Nuclei Profiling Tool for Drug Toxicity Screening. PLoS Comput. Biol..

[17] Xu Y., Dai Z., Chen F., Gao S., Pei J., Lai L. (2015). Deep Learning for Drug-Induced Liver Injury. J. Chem. Inf. Model..

[18] Taigman Y., Yang M., Ranzato M., Wolf L. DeepFace: Closing the Gap to Human-Level Performance in Face Verification. Proceedings of the 2014 IEEE Conference on Computer Vision and Pattern Recognition.

[19] Dong J., Wang N.-N., Yao Z.-J., Zhang L., Cheng Y., Ouyang D., Lu A.-P., Cao D.-S. (2018). ADMETlab: A Platform for Systematic ADMET Evaluation Based on a Comprehensively Collected ADMET Database. J. Cheminform..

[20] Kleinstreuer N.C., Karmaus A.L., Mansouri K., Allen D.G., Fitzpatrick J.M., Patlewicz G. (2018). Predictive Models for Acute Oral Systemic Toxicity: A Workshop to Bridge the Gap from Research to Regulation. Comput. Toxicol..

[21] Lim E., Pon A., Djoumbou Y., Knox C., Shrivastava S., Guo A., Neveu V., Wishart D. (2010). T3DB: A Comprehensively Annotated Database of Common Toxins and Their Targets. Nucleic Acids Res..

[22] Bradley A.P. (1997). The Use of the Area under the ROC Curve in the Evaluation of Machine Learning Algorithms. Pattern Recognit..

[23] Huang J., Ling C.X. (2005). Using AUC and Accuracy in Evaluating Learning Algorithms. IEEE Trans. Knowl. Data Eng..

[24] Banko M., Brill E. (2001). Scaling to Very Very Large Corpora for Natural Language Disambiguation. Proceedings of the 39th Annual Meeting on Association for Computational Linguistics.

[25] Sharma A.K., Srivastava G.N., Roy A., Sharma V.K. (2017). ToxiM: A Toxicity Prediction Tool for Small Molecules Developed Using Machine Learning and Chemoinformatics Approaches. Front. Pharmacol..

[26] Limbu S., Sivanesan D. (2022). Predicting Chemical Carcinogens Using a Hybrid Neural Network Deep Learning Method. Sensors.

[27] Batista G.E.A.P.A., Prati R.C., Monard M.C. (2004). A Study of the Behavior of Several Methods for Balancing Machine Learning Training Data. SIGKDD Explor. Newsl..

