# Supplementary material for: Predicting Dose-Range Chemical Toxicity using Novel Hybrid Deep Machine-Learning Method
Source: Toxics. 2022 Nov 18;10(11):706. doi: 10.3390/toxics10110706 (PMC9692315; doi:10.3390/toxics10110706)
Supplement: Supplementary file 1 [file toxics-10-00706-s001.zip › toxics-2003777-supplementary.pdf]

toxics MDPI

Supplementary Materials

Equation (S1). Equation to calculate the ensemble probability derived by Mayr et al[13].

$$\frac{\prod_{i=1}^{n} p(t = 1 \mid y_i)}{\prod_{i=1}^{n} p(t = 1 \mid y_i) + \prod_{i=1}^{n} p(t = 0 \mid y_i)}$$

Where, n is the number of models, y_i *is the prediction score by the model i,* $p(t = 1|y_i)$ *is the predicted probability for class 1 and* $p(t = 0|y_i)$ *is the predicted probability for class 0.*

Equation (S2). Equations to calculate the evaluation metrics.

$$Accuracy = \frac{TP + TN}{TP + TN + FN + FP} \times 100$$

$$Sensitivity(truepositiverate) = \frac{TP}{TP + FN} \times 100$$

$$Specificity(truenegativerate) = \frac{TN}{TN + FP} \times 100$$

$$Precision = \frac{TP}{TP + FP} \times 100$$

Where, TP = True Positive, TN = True Negative, FP = False Positive, and FN = False Negative.

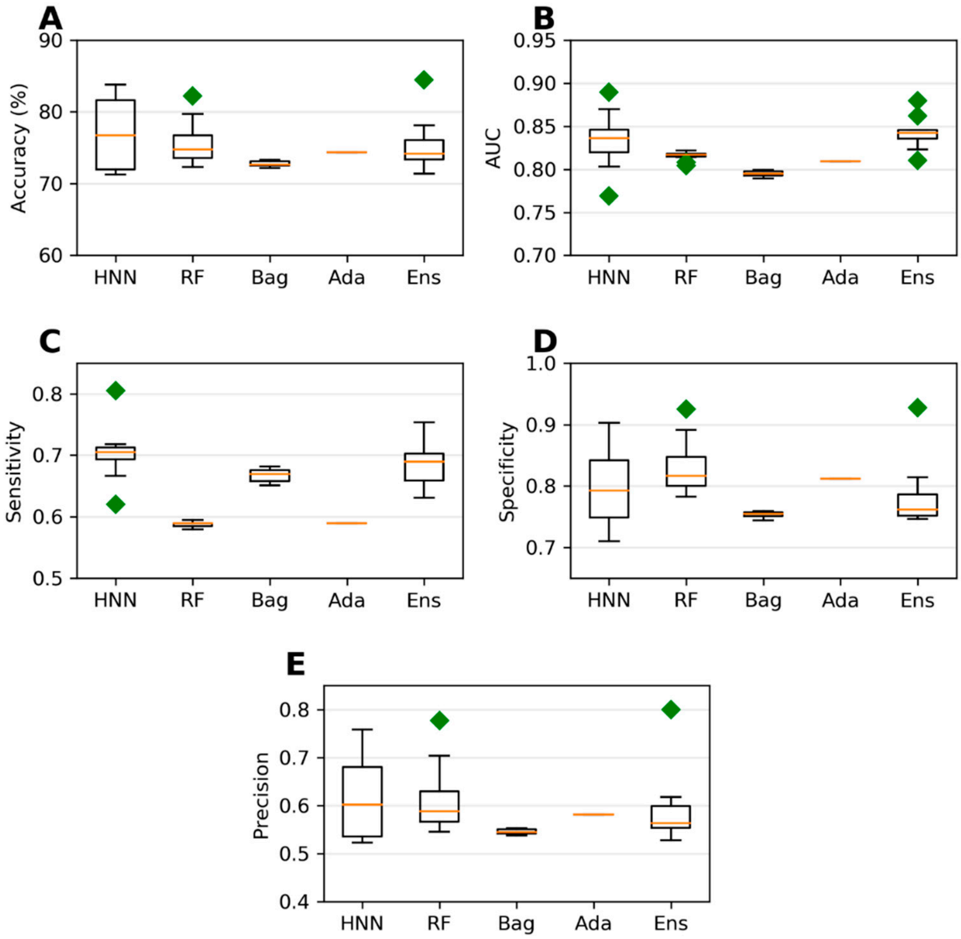

Figure S1. (A) Accuracy, (B) AUC, (C) Sensitivity, (D) Specificity and (E) Precision for the T3DB external validation dataset predicted by HNN-Tox, RF, Bagging, AdaBoost and the Ensemble methods to validate the models built on the ChemIDplus Oral data.

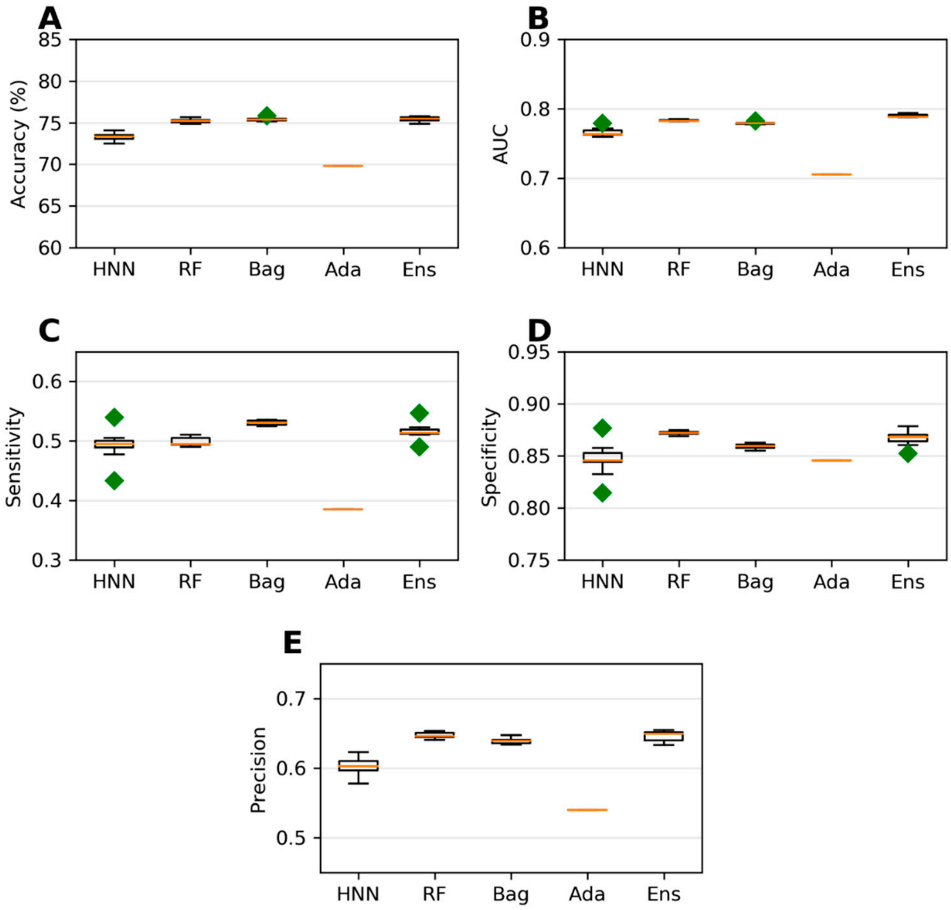

Figure S2. (A) Accuracy, (B) AUC, (C) Sensitivity, (D) Specificity and (E) Precision for the external validation dataset obtained from NTP, predicted by the HNN-Tox, RF, Bagging, AdaBoost and the Ensemble methods to validate the models built on ChemIDplus Oral data.

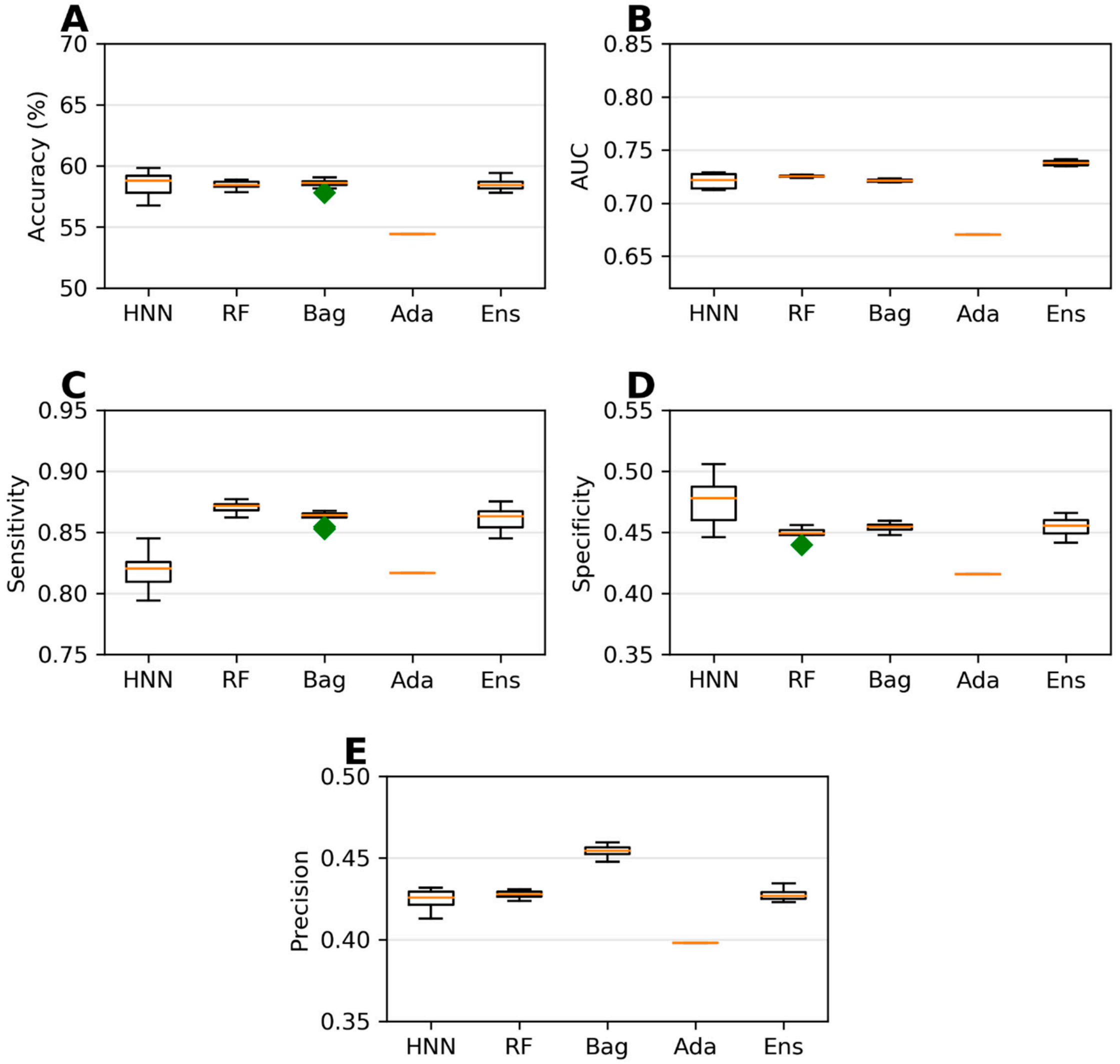

Figure S3. (A) Accuracy, (B) AUC, (C) Sensitivity, (D) Specificity and (E) Precision for the NTP external validation dataset predicted by the HNN-Tox, RF, Bagging, AdaBoost and the Ensemble methods to validate the models built on ChemIDplus IP/IV/Sub/Oral data.

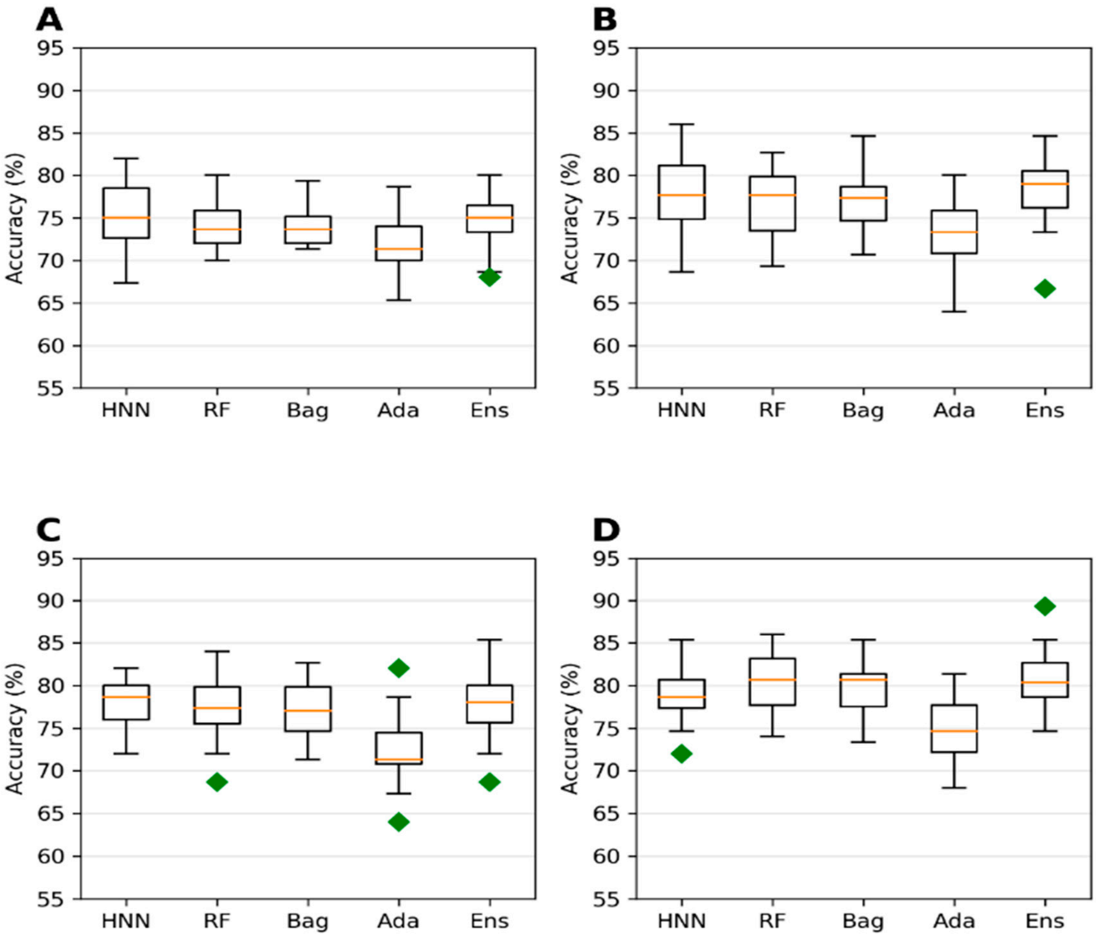

Figure S4. Accuracy of the Toxins data with IP, IV, Subcutaneous and Oral route of exposure with various LD_{50} cutoff (A) 250 mg/kg, (B) 500 mg/kg, (C) 750 mg/kg and (D) 1000 mg/kg predicted by the HNN-Tox, RF, Bagging, AdaBoost and the Ensemble methods.

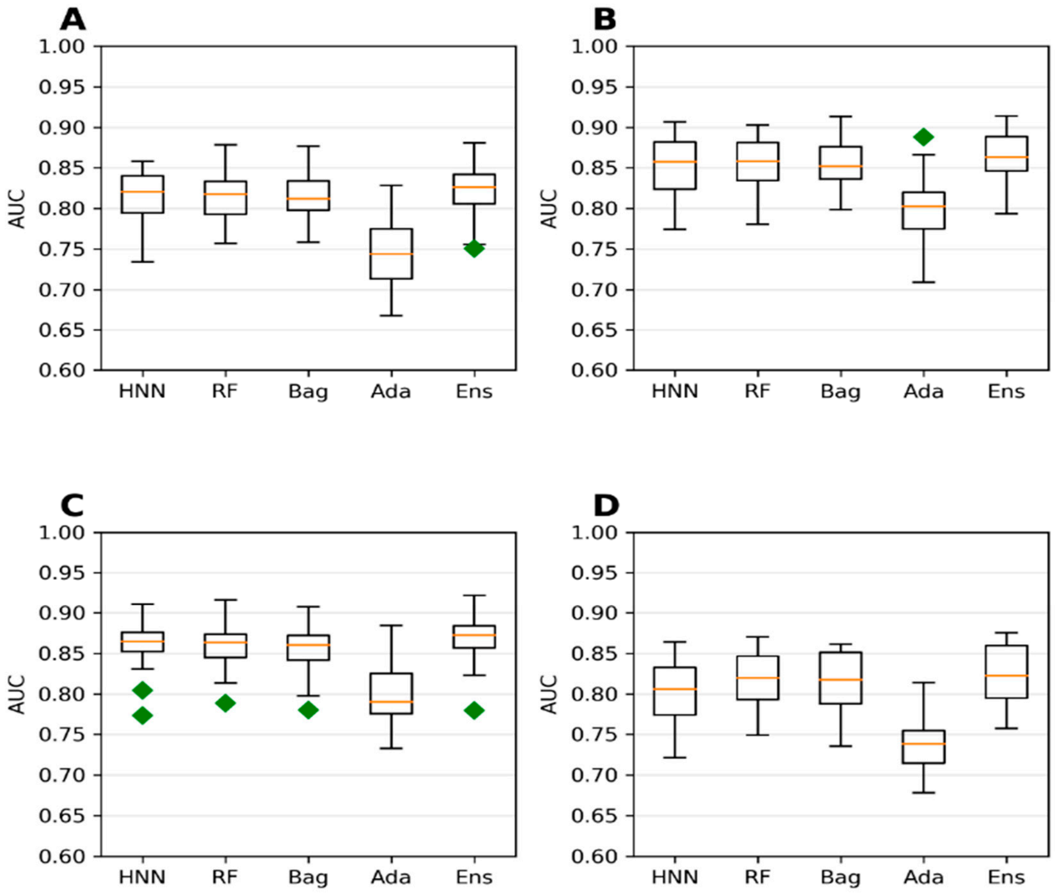

Figure S5. AUC for Toxins data obtained with IP, IV, Subcutaneous and Oral route of exposure with various LD_{50} cutoff (A) 250 mg/kg, (B) 500 mg/kg, (C) 750 mg/kg and (D) 1000 mg/kg predicted by the HNN-Tox, RF, Bagging, AdaBoost and the Ensemble methods.

Table S1. T3DB Toxins data annotated with different LD_{50} threshold values.

LD_{50} threshold (mg/kg)	*T3DB Toxins data*	
	62 descriptors (778 chemicals)	
	Toxic	*Nontoxic*
< 1000	*632*	*146*
< 750	*404*	*374*
< 500	*363*	*415*
< 250	*285*	*493*

Table S2. T3DB Toxins IP/IV/Subcutaneous/Oral route of exposure data annotated with different LD_{50} threshold values.

LD_{50} threshold (mg/kg)	*Oral*		*IP, IV, Subcutaneous*	
	62 descriptors (687 chemicals)		*62 descriptors (752 chemicals)*	
	Toxic	*Nontoxic*	*Toxic*	*Nontoxic*
< 1000	*285*	*402*	*573*	*179*
< 750	*262*	*425*	*345*	*407*
< 500	*225*	*462*	*307*	*445*
< 250	*157*	*530*	*238*	*514*

Table S3. Animal toxicity data from the EPA annotated with different LD_{50} threshold values.

LD_{50} threshold (mg/kg)	*EPA's Animal toxicity data 62 descriptors (427 chemicals)*	
	Toxic	*Nontoxic*
< 1000	418	9
< 750	418	9
< 500	412	15
< 250	404	23

Table S4. Combined data after merging animal toxicity data from the EPA (Table S1) and toxins data from the T3DB (Table S3) annotated with different LD_{50} threshold values.

LD_{50} threshold (mg/kg)	*EPA's Animal Toxicity data + T3DB data 62 descriptors (1054 chemicals)*	
	Toxic	*Nontoxic*
< 1000	941	113
< 750	720	334
< 500	687	367
< 250	623	431

Table S5. Distribution of Toxins Oral data among four classes in the multiclass classification.

LD_{50} Threshold (mg/kg)	*Class*	*No. of Chemicals*
LD50 < 50	3	68
50≤ LD50 < 500	2	157
500 ≤ LD50 < 1000	1	60
LD50 ≥ 1000	0	402
